# Universality of ultrasonic attenuation coefficient of amorphous systems at low temperatures

**DOI:** 10.1038/s41598-022-06589-7

**Published:** 2022-02-17

**Authors:** Pragya Shukla

**Affiliations:** Department of Physics, Indian Institute of Technology, Kharagpur, 721302 India

**Keywords:** Statistical physics, Condensed-matter physics

## Abstract

The competition between unretarded dispersion interactions between molecules prevailing at medium range order length scales and their phonon induced coupling at larger scales leads to appearance of nano-scale sub structures in amorphous systems. The complexity of intermolecular interactions gives rise to randomization of their operators. Based on a random matrix modelling of the Hamiltonian and its linear response to an external strain field, we show that the ultrasonic attenuation coefficient can be expressed as a ratio of two crucial length-scales related to molecular dynamics. A nearly constant value of the ratio for a wide range of materials then provides a theoretical explanation of the experimentally observed qualitative universality of the ultrasonic attenuation coefficient at low temperatures.

## Introduction

Experiments on thermal conductivity and acoustic attenuation in past have revealed an striking physical property of amorphous systems at low temperatures i.e. the universality of the internal friction $Q^{-1}$, defined in terms of the ratio of wavelength $\lambda$ of the elastic wave to its mean free path *l* and a standard measure of the ultrasonic attenuation in the medium^1^. For $T = 0.1 \rightarrow 10\, \text {K}$, $Q^{-1}(\omega ; T)$ is found to be nearly independent of temperature *T* as well as measuring frequency $\omega$. The magnitude of $Q^{-1}$ not only lies within about a factor of 20 for all glasses but is also very small (around $\sim 10^{-4}$), indicating long (short) mean free paths at small (large) phonon frequencies.

Previous attempts to explain this behaviour were based on an assumed existence of the defects modeled as tunnelling two level systems (TTLS)^2^. Although successful in explaining many glass anomalies, the original TTLS model^3–6^ suffered many drawbacks^7–9^ (besides experimental lack of evidence supporting their existence in most glasses). This encouraged attempts for improvements of the model by incorporating a phonon-TTLS interaction^10^, presence of TLS alongwith quasi-harmonic oscillators^11^ as well as considerations of several new theories; (extensive research on this topic during previous decades renders it impossible to list all but a few leading to new theoretical developments e.g.^3–6,8,9,11–22^).

In context of the acoustic attenuation, an important direction was taken in a recent theory of coupled generic blocks with a phonon-mediated interaction of type $1/r^3$ with *r* as the separation between blocks^9,23^. A renormalisation approach used in Ref.^23^ rendered the information regarding the behavior of a single generic block unnecessary and provided useful insights regarding the universality at macroscopic scales. Although the theory was later on applied successfully to explain another glass-universality, namely, Meissner–Berret ratio^24^, it has still left many questions unanswered e.g. how the block type structure appears, what is the effect of the intra-block forces over the inter-block ones, whether the universality is an emergent phenomenon occurring only at large scales or it also occurs at microscopic scales i.e. for a single block; (for example, the study^23^ does not provide any information about the attenuation coefficient for a basic block). An answer to these questions is pertinent to understand the physical origin of universalities which motivates the present work.

Based on the nature of chemical bonding, the physics of solids is expected to vary at microscopic length scales. Contrary to other low temperature properties, however the ratio $\lambda /l$ is found to be universal not only for glasses (with only few exceptions in some thin films) but for a huge class of materials different from them at large length scales e.g. disordered crystals, poly-crystals, some quasi crystals etc^1^. Furthermore the irradiation experiments on crystalline silicon for a wide range of radiation doses indicate the sound properties of the irradiated samples similar to glasses. Another universality not confined only to glasses but applicable to many liquids too is that of excess vibrational density of states which can not be explained based on the phonon contributions only^25^. These universalities therefore seem to originate from more fundamental considerations, shared by both amorphous as well as disordered crystalline materials, with lack of long-range order not the cause of the low-energy excitations, and applicable not only for macroscopic sizes but also at microscopic scales (e.g. see^26,27^ also in this context). This motivated us in Ref.^28^ to consider the intermolecular interactions, more specifically Vanderwaals (VW) forces among the molecules within a block as the basis for the behavior; it is important to emphasise here that VW forces among molecules are always present in all condensed phases and therefore are the natural candidates to decipher the experimentally observed universality.

Our primary focus in the present work is to seek the physical origin of the weak attenuation of the sound waves in amorphous systems. For this purpose, it is necessary to first identify the local structures which respond to an external strain field by collective vibrations of molecules. But phonons in a perfect harmonic dielectric crystal are free of interactions, leading to a sound wave travel unattenuated. To understand long mean free paths in glasses at low frequencies, this intuitively suggest to seek for ordered structure, at least locally, and repeated almost periodically. The structure related to medium range order (MRO) in glasses seem to be playing the relevant role. (Note the glasses also have short range order but that is governed by covalent bonds which are quite rigid to undergo deformation by a weak strain field. Further the role of the molecular clusters or structural correlations was proposed in past too e.g. in Refs.^25,29–31^) but it was not very well-defined^32^). As discussed in Ref.^33^, the size of the basic block indeed turns out to be that of the length scale associated with medium range order (Note the peculiarity of role played by MRO in context of acoustic modes was mentioned in Ref.^32,34^ too; the study^34^ indicated that the continuum approximation for the medium, necessary for Debye formulation, breaks down for acoustic modes with wavelength less than MRO). The combination of many such blocks can then provide required periodicity and their long-range interaction result in attenuation only at long length scales. Our theory of coupled blocks is therefore based on two main types of interactions, dominant at different spatial scales; a competition between them governs the block-size and also gives rise to an inter-connected block structure, with phonon mediated coupling of their stress fields. This in turn leads to formulation of the attenuation in terms of the stress–stress correlations among basic blocks and their density of the states. As discussed later, both of them can be expressed in terms of the molecular properties which finally leads to a constant, system-independent average value of $Q^{-1}$.

The paper is organized as follows. The theory of an amorphous system of macroscopic size as a collection of sub-structures coupled with each other via an inverse-cube phonon mediated interaction is discussed in detail in Refs.^23,24^; this is briefly reviewed in “Super block: phonon mediated coupling of basic blocks” section, with macroscopic solid referred as the super block and the sub-structures referred as the basic blocks. Note the present work differs from Refs.^23,24^ in context of the basic blocks details; the latter appear, in our theory, as a result of VW interactions among molecules prevailing at nano-scales^28^. The theory is used in “Ultrasonic attenuation coefficient: relation with stress matrix” section to relate the $\langle Q^{-1} \rangle$ of the super block to the stress-stress correlations of the basic blocks, their bulk density of states $\rho _e$ and volume $\Omega _b$; here 〈 〉 refers to the ensemble as well as spectral average. $\rho _e$ depends on a parameter *b*, referred as “bulk spectral parameter” and derived in Ref.^28^ in terms of the molecular parameters. This along with $\Omega _b$ leads to dependence of $Q^{-1}$ on the length-ratio $R_0/ R_v$ with $R_0$ as the linear size of the basic block and $R_v$ as the distance between two nearest neighbor molecules mutually interacting by unretarded dispersion forces. A theoretical analysis of the ratio $R_0/R_v$ has indicated it to be a system-independent constant for amorphous systems (supported by data based on 18 glasses)^33^. In “[Sec Sec10]” section, we express $\langle Q^{-1}\rangle$ in terms of $R_0/ R_v$ and thereby theoretically prove its quantitative universality. It can however be calculated directly from the molecular properties too; as discussed in “Comparison with experimental data” section, a good agreement of the results so obtained for 18 non-metallic glasses with experimental values not only lends credence to our theory of blocks but also provides an indirect route to reconfirm the universality of the ratio $R_v/R_0$. Note the 18 glasses chosen for comparison here are same as those used in Ref.^35^. A discussion of physical insights provided by our approach, brief comparison with other theories and approximations are outlined in “Discussion” section. We conclude in “Conclusion” section with a summary of our main ideas and results.

## Super block: phonon mediated coupling of basic blocks

The order at atomic dimensions in an amorphous solid is system dependent; it is sensitive to the nature of chemical bonding. The intuition suggests the universal properties to originate from the interactions which appear at length scales at which the solid manifests no system-dependence. It is therefore relevant to seek and identify the sub-units in the super block structure which give rise to such interactions. For this purpose, let us first express the Hamiltonian *H* of the amorphous solid of volume $\Omega$ as the sum over intra-molecular interactions as well as inter-molecular ones

1 $$\begin{aligned} H= \sum _k h_k(\mathbf{r}_k) + {1 \over 2} \sum _{k,l} {\mathcal U}(|\mathbf{r}_k - \mathbf{r}_l |), \end{aligned}$$

with $h_k$ as the Hamiltonian of the kth molecule at position $\mathbf{r}_k$ and ${{\mathcal {U}}}$ as an inter-molecular interaction with arbitrary range $r_0$. Assuming that all the relevant many body states are “localized”, in the sense that the probability density for finding a given molecule “*k*” is “concentrated” (as defined by its mean square radius) in a region of finite radius *l* around some point $\mathbf{r}_k$, it is possible to define a 3*D* lattice (grid of points) $\mathbf{R}_{\alpha }$ with spacing $d \gg r_0$ such that the molecule “k” is associated with that lattice point $\mathbf{R}_{\alpha }$ which is closest to $\mathbf{R}_{k}$. The association is fixed, is insensitive to the dynamics and corresponds to representation of the solid by 3-dimensional blocks of linear size $R_0$, with their centers at lattice points $\mathbf{R}_{\alpha }$. The Hamiltonian *H* can then be reorganised as a sum over basic block Hamiltonians and the interactions between molecules on different blocks

2 $$\begin{aligned} H= \sum _s {{\mathcal {H}}}^{(s)} + {1 \over 2} \sum _{s,t} \sum _{k \in s, l \in t} {{\mathcal {U}}}(|\mathbf{r}_k - \mathbf{r}_l |), \end{aligned}$$

where ${{\mathcal {H}}}^{(s)}$ is the Hamiltonian of a basic block labeled “$s$”, basically sum over the molecular interactions within the block: ${{\mathcal {H}}}^{(s)} = \sum _{k \in s} h_k(\mathbf{r}_k) + {1 \over 2} \sum _{k,l \in s} {{\mathcal {U}}}(|\mathbf{r}_k - \mathbf{r}_l |)$. As mentioned below, the molecules interactions appearing in 2nd term in Eq. (2) rearrange themselves collectively and results in emergence of coupled stress fields of the blocks. The number *g* and volume $\Omega _b$ of these blocks can be determined by analysing the competition between inter-molecular forces with emerging forces i.e phonon mediated coupling: $g=\Omega /\Omega _b$ with $\Omega _b \sim R_0^3$. The statistical behavior of the Hamiltonian ${{\mathcal {H}}}$ is discussed in detail in Ref.^28^.

To analyze the ultrasonic attenuation in glasses, we first need to analyze the response of ${{\mathcal {H}}}$ to an external strain field.

### Perturbed Hamiltonian of a basic block

In presence of an external strain field, the molecules in a glass block are displaced from their equilibrium position and their interactions with those in surrounding blocks give rise to a stress field distributed over the block. Let $u(\mathbf{r})$ be the displacement, relative to some arbitrary reference frame, of the matter at point $\mathbf{r}$, the elastic strain tensor can then be defined as

3 $$\begin{aligned} e_{\alpha \beta }(\mathbf{r},t)& = {1 \over 2} \left( {\partial u_{\alpha } \over \partial x_{\beta }} + {\partial u_{\beta } \over \partial x_{\alpha }} \right) , \end{aligned}$$

with subscripts $\alpha , \beta$ referring to the tensor-components.

This gives rise to stress in the block which can in general have both elastic as well as inelastic components. The perturbed Hamiltonian $H_{pt}$ of the basic block, labeled “*s*” can then be written as a sum over elastic and inelastic contributions

4 $$\begin{aligned} { {\mathcal {H}}}_{pt}^{(s)} = {{\mathcal {H}}}_{pt,ph}^{(s)} + {\mathcal H}_{pt,nph}^{(s)}. \end{aligned}$$

Each of these parts can further be expanded as a Taylor’ series around unperturbed block Hamiltonian ${{\mathcal {H}}}_x$ in terms of strain $e_{\alpha \beta }$ in long wavelength limit (where the subscript “*x*” refers to the elastic (“$x=ph$”) and inelastic parts (“$x=nph$”) respectively):

5 $$\begin{aligned} {{\mathcal {H}}}^{(s)}_{pt, x}(t) = {{\mathcal {H}}}_{x}^{(s)} + \int \text{d}{} \mathbf{r} \; e_{\alpha \beta }(\mathbf{r},t) \; {\Gamma }^{(s)}_{\alpha \beta ; x}(\mathbf{r}) + O(e_{\alpha \beta }^2), \end{aligned}$$

with $\Gamma ^{(s)}_{\alpha \beta ; x}(\mathbf{r})$ as the stress tensor; as clear from above $\Gamma ^{(s)}_{\alpha \beta ; x}(\mathbf{r}) = {\partial {{\mathcal {H}}}^{(s)}_{pt, x} \over \partial e_{\alpha \beta } }$. Here the retaintion of terms in the Taylor series expansion only up to first order in $e_{\alpha \beta }$ assumes the small strength of the strain field perturbation relatively to the unperturbed block Hamiltonian.

While a quantitative measure of the exact strength of the strain filed e.g. its weakness is not really needed for our analysis and is therefore beyond the purview of the present work, qualitatively the validity of Eq. (5) refers to a strength which permits (i) the treament of the block response to the external strain field within linear response theory (discussed in “Non-phonon linear response function” and in detail in Ref.^23^). We note the latter theory is used extensively for the mathematical formulation in this work, (ii) ignoring the rotation of the block etc under strain field, (iii) replacement of the distributed stress field within the block of volume $\Omega _b$ by an average field, acting from the centre of mass of the block: $\int _{\Omega _b} \; \text{d}{} \mathbf{r} \; \Gamma ^{(s)}_{\alpha \beta } (\mathbf{r}) = \Gamma ^{(s)}_{\alpha \beta }$ (based on the assumption of the isotropy and the small block-size).

The perturbed Hamiltonian of the basic block can then be approximated as

6 $$\begin{aligned} { {\mathcal {H}}}_{pt;x}^{(s)} = {{\mathcal {H}}}^{(s)}_x + \sum _{\alpha \beta } e^{(s)}_{\alpha \beta } \; {\Gamma }^{(s)}_{\alpha \beta ; x}, \end{aligned}$$

with $e^{(s)}_{\alpha \beta } (t)$ referring to the phonon strain field $e_{\alpha \beta } (\mathbf{r}, t)$ at the *s*-th block.

### Super block Hamiltonian

The super block consists of *g* basic blocks, perturbed by mutual interaction. To proceed further, it is useful to separate its Hamiltonian *H* into phononic and non-phonon contributions (referred by subscripts “$ph$” and “$nph$” respectively): $H= H_{ph} + H_{nph}$^9^. The contribution of elastic part $H_{ph}$ to the ultrasonic attenuation in glass super block at temperatures $T < 1 K$ is negligible. We therefore need to consider the contribution from the inelastic part $H_{ nph}$ only; to reduce notational complexity, henceforth, the subscripts “$nph$” will be suppressed and the notations $H, {{\mathcal {H}}}^{(s)}_{pt}, \Gamma ^{(s)}$ etc will be used for $H_{nph}, {\mathcal H}^{(s)}_{pt;nph}, \Gamma ^{(s)}_{nph}$ respectively.

As the strain tensor $e_{\alpha \beta }$ contains a contribution from the phonon field, the exchange of virtual phonons will give rise to an effective (“RKKY”-type) coupling between the stress tensors of any two block-pairs. Let $\Gamma ^{(s)}_{\gamma \delta } (\mathbf{r})$ be the stress tensor at point $\mathbf{r}$ of the basic block ”s”. The interaction $V_{st}$ between the blocks “s” and “t ” can be given as^23^

7 $$\begin{aligned} V_{st}&= {1\over 4 \pi \rho _m c_t^2} \; \int _s \text{d}{} \mathbf{r} \; \int _{t} \text{d}{} \mathbf{r'} \; \sum _{te} \; {\kappa ^{(st)}_{\alpha \beta \gamma \delta } \over | \; \mathbf{r}-\mathbf{r'} \; |^3 }. \; \; \Gamma ^{(s)}_{\alpha \beta } (\mathbf{r})\otimes \; \Gamma ^{(t)}_{\gamma \delta } (\mathbf{r'}), \end{aligned}$$

with $\rho _m$ as the mass-density and $c_a$, as the longitudinal ($al$) or transverse ($a \equiv t$) sound velocity in the super block. Here the subscripts $\alpha \beta \gamma \delta$ refer to the tensor components and the symbol $\sum _{te}$ refers to a sum over all tensor components: $\sum _{te} \equiv \sum _{\alpha \beta \gamma \delta }$. The directional dependence of the interaction is represented by $\kappa ^{(st)}_{\alpha \beta \gamma \delta }=\kappa ^{(st)}(\theta , \phi )$; it is assumed to depend only on the relative orientation ($\theta , \phi$) of the block-pairs and is independent from their relative separation^24^:

8 $$\begin{aligned} \kappa ^{(st)}_{ijkl}&= - \left( \delta _{jl} - 3 n_j n_l \right) \delta _{ik} + \nu _2 \;\left[ - (\delta _{ij} \delta _{kl} + \delta _{ik} \delta _{jl} + \delta _{il} \delta _{jk}) + \right. \nonumber \\&+ \left. 3 \; \left( n_j n_l \delta _{ik} + n_j n_k \delta _{il} + n_i n_k \delta _{jl} + n_i n_l \delta _{jk} + n_i n_j \delta _{kl} + n_k n_l \delta _{ij} \right) - 15 \; \sum _{ijkl} \; n_i n_j n_k n_l \right] , \end{aligned}$$

where $\nu _2= \left( 1-{c_t^2 \over c_l^2}\right)$ and $\mathbf{n} = n_1 {{\hat{i}}} + n_2 {{\hat{j}}} + n_3 {{\hat{k}}}$ is the unit vector along the direction of position vector $\mathbf{r-r'}$. Again assuming the isotropy and the small block-size, the interaction between various points of the block-pairs can be replaced by the average interaction between their centers $\mathbf{R_s}$ and $\mathbf{R_t}$. The phonon mediate coupling between the blocks can then be approximated as^23,24^

9 $$\begin{aligned} V_{st}&= {1\over 4 \pi \rho _m c_t^2} \; \sum _{\alpha \beta \gamma \delta } \; { \kappa ^{(st)}_{\alpha \beta \gamma \delta } \over | \; \mathbf{R_s}-\mathbf{R_{t}} \; |^{3} } \; \; \Gamma ^{(s)}_{\alpha \beta } \otimes \; \Gamma ^{(t)}_{\gamma \delta }. \end{aligned}$$

Due to the above emerging interactions at large length scales, the super block Hamiltonian in Eq. (2) is not just a sum over basic block Hamiltonians but also includes their phonon mediated coupling.

Equation (9) describes an emerging interaction at large length scales. The Hamiltonian of the super block in Eq. (2) can now be rearranged as a sum over those of the basic blocks as well as their phonon mediated coupling. In absence of external strain field, the non-phonon part of *H* can be rewritten as

10 $$\begin{aligned} H=H_0 +V, \end{aligned}$$

with $H_0$ as a sum over non-phonon part of the unperturbed basic block Hamiltonians, $H_0 = \sum _{s=1}^g \; {{\mathcal {H}}}^{(s)}$, and, *V* as the net pair-wise interaction among blocks: $V=\sum _{s,t; s\not =t} V_{st}$ where $\sum _{s,t}$ implies the sum over all basic blocks. The presence of a weak external strain field perturbs the basic blocks and thereby *H*. The non-phonon part of the perturbed Hamiltonian $H_{pt}$ can be written as^23,24^

11 $$\begin{aligned} H_{pt} = H + \sum _{s=1}^g \sum _{\alpha \beta } e_{\alpha \beta }^{(s)} \; \Gamma _{\alpha \beta }^{(s)} = H + \sum _{\alpha \beta } e_{\alpha \beta } \; \Gamma _{\alpha \beta }, \end{aligned}$$

where the 2nd equality follows by assuming the same strain operator for all blocks $e_{\alpha \beta }^{(s)} \approx e_{\alpha \beta }$ and writing $\Gamma _{\alpha \beta } = \sum _{s=1}^g \Gamma _{\alpha \beta }^{(s)}$. (Note, as discussed in Ref.^24^, the total Hamiltonian for the super block contains two additional terms besides *V* (see Eq. (2.21) in Ref.^24^) but their ensemble averaged contribution is negligible. Alternatively it can also be absorbed by redefining stress operators).

## Ultrasonic attenuation coefficient: relation with stress matrix

In general, a wave propagating through a natural medium undergoes attenuation of its intensity with distance due to spreading, scattering as well absorption. For example, the change of amplitude in case of an attenuating plane wave can be expressed as $\Phi (x)=\Phi (0) \; \text{e}^{-\alpha x}$ with $\Phi (x)$ as the amplitude at position *x* and $\alpha$ as the “attenuation coefficient”. The latter in general is a function of frequency $\omega$, wave speed and quality factor of the medium and its measurement leads to the mean free path *l* of the wave $l= \alpha ^{-1}$. For comparison of experiments on different materials, it is useful to a define the dimensionless ultrasonic attenuation coefficient $Q_{a}^{-1}(\omega )$ for a wave of frequency $\nu$ and wavelength $\lambda$^1,24^:

12 $$\begin{aligned} Q_{a}^{-1} ={1 \over 2 \pi ^2 } {c_a\over \nu } \; \alpha = {1 \over 2 \pi ^2 } \; {\lambda \over l}, \end{aligned}$$

with $c_a$ as the speed of acoustic wave in the longitudinal (with $a \equiv l$) or transverse direction ($a \equiv t$). Here we note that the above definition is different from the one given in Ref.^1^ by a constant: $Q_{a,pohl}^{-1} = \pi \; Q_{a}^{-1}$. Further the quality factor of the medium corresponds to inverse of $Q_{a}^{-1}$; with former often defined as the “inverse internal friction”, this in turn leads to $Q_{a}^{-1}$ often referred as “intrenal friction” too.

Consider the attenuation of acoustic waves in a glass super block with its Hamiltonian *H* given by Eq. (11). Assuming the coupling between phonon and non-phonon degrees of freedom a weak perturbation on the phonon dynamics, $Q_{a}^{-1}(\omega )$ can be expressed as^24^

13 $$\begin{aligned} Q_{a}^{-1}(\omega ) = ( \pi \; \rho _m \; c_a^2)^{-1} \; \text{Im} \; \chi _a(\omega ), \end{aligned}$$

with $\rho _m$ as the mass-density of the material. Here $\chi _{l,t}(\omega )$, referred as the longitudinal or transverse response function, are the measures of the linear response of the basic blocks to external strain field and can be defined as follows; (see^23^ for a detailed discussion).

### Non-phonon linear response function

Consider the linear response of a basic block, labeled as “$s$”, to an external strain field $e_{ij}(\mathbf{r}, t)= e_{ij} \; exp[i(\mathbf{q.r} - \omega t)]$ with $e_{ij}$ real but infinitesimal. The perturbed Hamiltonian is given by Eq. (6) with corresponding stress-field given as $\Gamma ^{(s)}_{ij}(\mathbf{r}, t) = {{\hat{\Gamma }}^{(s)}_{ij}} \; exp[i(\mathbf{q.r} - \omega t)]$ where $\langle \Gamma ^{(s)}_{ij}\rangle$ is in general complex.

The complex response function or the susceptibility for a basic block can then be defined as

14 $$\begin{aligned} \chi ^{(s)}_{\alpha \beta \gamma \delta }(\mathbf{q}, \omega ) \equiv \frac{1}{\Omega _b} \; {\partial \; {{\hat{\Gamma }}^{(s)}_{\alpha \beta } (\mathbf{q}, \omega )} \over \partial \; e_{\gamma \delta }}. \end{aligned}$$

Here in general the variable $\mathbf{q}$ and $\omega$ are independent variables. But as our interest is in values of *q* close to $\omega /c_{l,t}$, $\chi$ will henceforth be written as a function of $\omega$ only^23^.

The imaginary part of $\chi ^{(s)}(\omega )$ can be written in the representation in which unperturbed basic block Hamiltonian ${{\mathcal {H}}}^{(s)}$ is diagonal (later referred as non-interacting or NI basis). Let $|{m_s} \rangle$, $m_s = 1\rightarrow N$ be the many body eigenstate of ${{\mathcal {H}}}^{(s)}$ with energy $e_m$, then

15 $$\begin{aligned} \text{Im} \; \chi ^{(s)}_{\alpha \beta \gamma \delta } (\omega )& = {(1-\text{e}^{-\beta \omega })\over Z}\; \sum _m \; e^{-\beta e_m} \; \chi ^{(m,s)}_{\alpha \beta ;\gamma \delta }(\omega ), \end{aligned}$$

with Z as the partition function. Here to simplify presentation, we set $\hbar =1$. Further

16 $$\begin{aligned} \chi ^{(m,s)}_{\alpha \beta \gamma \delta }(\omega ) = \frac{\pi }{\Omega _b} \; \sum _{n=1}^N \; \Gamma ^{(s)}_{\alpha \beta ; mn} \; \Gamma ^{(s)}_{\gamma \delta ; n m} \;\; \delta (e_n-e_m - \omega ), \end{aligned}$$

with $\Gamma ^{(s)}_{\alpha \beta ; kl}$ as the matrix element of the stress-tensor in the NI basis: $\Gamma ^{(s)}_{\alpha \beta ; kl} = \langle {k_s} | \; \Gamma ^{(s)}_{\alpha \beta } \; | {l_s} \rangle$.

Substitution of Eqs. (15) and (16) in Eq. (13) leads to a frequency dependent formulation of $Q_a^{-1}$ and thereby a dispersion of sound velocities (following from the Kramer–Kronig relations). However, as mentioned in “Introduction” section, extensive experimental studies during last few decades have clearly indicated a nearly temperature as well as frequency independence of the attenuation coefficient for a wide range of amorphous systems at low temperatures; these observations are also confirmed by independent measurements of dispersion in sound velocities in the medium^36^. Another rather more puzzling feature revealed by experiments is almost quantitative universality of $Q^{-1}$ ( $\approx 10^{-4}$) for many amorphous materials); these experimental findings are reviewed in detail in Ref.^1^. As such a small value of attenuation, equivalently long mean free paths of the phonons, in the amorphous medium is intuitively not obvious, this has baffled the researchers for last many decades (e.g. see^31^), has been an intense area of research and is also the primary focus of the present work. Hereafter, our present analysis would be confined to a frequency averaged ultrasonic attenuation coefficient only. While a detailed understanding of the qualitative universality i.e. frequency independence of $Q^{-1}$ is also desirable, this requires a more detailed mathematical analysis of the stress–stress correlations and will be discussed in a subsequent work.

In general $\chi ^{(m)}_{\alpha \beta \gamma \delta }$ depends on the energy level $e_m$ and fluctuates over the spectrum. It is then useful to define the spectral averaged susceptibility over the *N*-level spectrum of the basic block

17 $$\begin{aligned} {\langle \chi ^{(s)}_{\alpha \beta \gamma \delta } \rangle _{\omega }}&= {1\over N \omega _c} \sum _{m=1}^N \int _0^{\omega _c} \; \chi ^{(m,s)}_{\alpha \beta \gamma \delta }(\omega -e_m) \; \text{d}\omega , \end{aligned}$$

where $\omega _c$ is the bulk spectrum width of the basic block with $\langle . \rangle _{\omega }$ implying a spectral averaging.

Furthermore the fluctuations of $\Gamma ^{(s)}_{\alpha \beta ; kl}$ as well as those of the energy levels over the ensemble also influence $\chi ^{(m,s)}_{\alpha \beta \gamma \delta }(\omega )$ and it is appropriate to consider its ensemble average $\langle \chi ^{(m,s)}_{\alpha \beta \gamma \delta }(\omega ) \rangle _e$ too. Assuming isotropy, rotationally invariance of the basic block (as its linear size $L \gg a$ with *a* as the atomic length scale), all $3^8$ components of response function can further be expressed in terms of the transverse and longitudinal response^24^:

18 $$\begin{aligned} \langle \chi ^{(s)}_{\alpha \beta \gamma \delta }(\omega ) \rangle _{e, \omega }&= \left( q_c \; \delta _{\alpha \beta } \delta _{\gamma \delta } + \delta _{\alpha \gamma } \delta _{\beta \delta } + \delta _{\alpha \delta } \delta _{\beta \gamma } \right) \; \langle \chi ^{(s)}_t \rangle _{e, \omega }, \end{aligned}$$

where $q_c={\langle \chi ^{(s)}_l\rangle _{e,\omega } \over \langle \chi ^{(s)}_t\rangle _{e,\omega }} - 2$ along with $\langle . \rangle _e$ implying an ensemble averaging, $\langle . \rangle _{e, \omega }$ an averaging over both $\omega$ as well as ensemble.

The relations given in Eq. (14) to Eq. (18) are applicable for a basic block of volume $\omega _b$. Following similar forms of Eqs. (6) and (11), these can be generalized for the susceptibility $\langle \chi _a\rangle ^{sup}_{e,\omega }$ of a super block. This follows by dropping the superscript $``s''$ and with replacements $\Omega _b \rightarrow \Omega$, $N \rightarrow N^g, \omega _c \rightarrow W_c, e_n \rightarrow E_n$ in Eq. (14) to Eq. (18); note here $E_m$ refers to a many body energy level of *H* (defined in Eq. (10).

### Relation between $Q_{a}^{-1}$ and stress-correlations

#### For basic block

Due to disorder beyond atomic scales, a typical matrix element of the stress tensor of a basic block fluctuates over the ensemble and can be both positive as well as negative. This implies $\langle \Gamma ^{(s)}_{\alpha \beta ; kl} \rangle _e =0$. Further, at temperature $T =0$, the spectral averaging (defined in Eq. (17)) of Eq. (16) followed by an ensemble averaging leads to the stress-stress correlation of the basic block

19 $$\begin{aligned} \sum _{m, n=1}^N \langle \Gamma ^{(s)}_{\alpha \beta ; mn} \; \Gamma ^{(s)}_{\gamma \delta ; n m } \rangle _{e} ={ N \omega _c \; \Omega _b \over \pi } \left( q_c \; \delta _{\alpha \beta } \delta _{\gamma \delta } + \delta _{\alpha \gamma } \delta _{\beta \delta } + \delta _{\alpha \delta } \delta _{\beta \gamma } \right) \; \langle \text{Im} \; \chi ^{(s)}_t \rangle _{e.\omega }, \end{aligned}$$

where $\langle \text{Im} \; \chi ^{(s)}_t \rangle _{e.\omega }$ is defined in Eq. (18).

The short-range order of atomic positions in the basic-block along with its small size suggests a homogeneous nature of many body interactions. The ensemble averaged matrix elements of $\Gamma ^{(s)}_{\alpha \beta }$, in the NI basis i.e. the eigenfunction basis of $H^{(s)}_0$, can then be assumed to be of almost same strength. (This is equivalent to say that, due to small size of block, stress can be assumed to be homogeneous i.e. of the same order everywhere in the block. This assumption therefore puts a constraint on our basic-block size). One can then write $\sum _{ m, n=1} \langle \Gamma ^{(s)}_{\alpha \beta ; mn} \; \Gamma ^{(s)}_{\gamma \delta ; n m } \rangle _{e} = N^2 \; \langle \Gamma ^{(s)}_{\alpha \beta ; mn} \; \Gamma ^{(s)}_{\gamma \delta ; n m } \rangle _{e}$. This on substitution in Eq. (19) leads to

20 $$\begin{aligned} \langle \Gamma ^{(s)}_{\alpha \alpha ; mn} \; \Gamma ^{(s) }_{\gamma \gamma ; mn} \rangle _{e}&= { \omega _c \; \Omega _b \over N \pi } \; \left[ q_c + \delta _{\alpha \gamma } \right] \; \langle \text{Im} \; \chi ^{(s)}_t(\omega ) \rangle _{e, \omega }, \end{aligned}$$

21 $$\begin{aligned} \langle \Gamma ^{(s)}_{\alpha \beta ; mn} \; \Gamma ^{(s) }_{\alpha \beta ; m n } \rangle _{e}& = \langle \Gamma ^{(s)}_{\alpha \beta ; mn} \; \Gamma ^{(s) }_{\beta \alpha ; m n} \rangle _{e} = { \omega _c \; \Omega _b \over N \pi } \;\langle \text{Im} \; \chi ^{(s)}_t(\omega ) \rangle _{e, \omega }\quad \alpha \not = \beta . \end{aligned}$$

Further using Eq. (13) in Eqs. (20) and (21), the correlations can be expressed in terms of the average ultrasonic absorption $\langle Q^{-1}_t(\omega ) \rangle _{e,\omega }$ of the basic block

22 $$\begin{aligned} \langle \Gamma ^{(s)}_{\alpha \alpha ; mn} \; \Gamma ^{(s) }_{\gamma \gamma ; mn} \rangle _{e}&= {N^{-1} \; \omega _c \; \rho _m \; c_t^2 \; \Omega _b } \; \langle Q^{-1}_t(\omega ) \rangle _{e,\omega } \; \delta _{\alpha \gamma }, \end{aligned}$$

23 $$\begin{aligned} \langle \Gamma ^{(s)}_{\alpha \beta ; mn} \; \Gamma ^{(s) }_{\alpha \beta ; m n } \rangle _{e}&= \langle \Gamma ^{(s)}_{\alpha \beta ; mn} \; \Gamma ^{(s) }_{\beta \alpha ; mn} \rangle _{e} = {N^{-1} \; \omega _c \; \rho _m \; c_a^2 \; \Omega _b} \langle Q^{-1}_t(\omega ) \rangle _{e,\omega }. \end{aligned}$$

Equation (23) can be rewritten in terms of the mean-square matrix element $\nu ^2 = \langle \left( \Gamma ^{(s)}_{\alpha \beta ; mn} \right) ^2 \rangle _{e}$

24 $$\begin{aligned} \langle Q_{a}^{-1} \rangle _{e, \omega }&= { N \; \nu ^2 \over \omega _c \; \rho _m \; c_a^2 \; \Omega _b } = { \gamma ^2 \over \omega _c \; \rho _m \; c_a^2 \; \Omega _b }, \end{aligned}$$

where $\gamma ^2 \equiv N^{-1} \; \text{Tr} (\Gamma ^{(s)}_{\alpha \beta })^2 = N \nu ^2$ is related to the coefficient of the phonon mediated coupling *V* between two basic blocks (which is of the form ${\gamma ^2\over 8 \pi \rho _m c^2 r^3}$, see Eq. (9)).

As discussed in Ref.^28^, the ensemble averaged density of the states which participate in these excitations, has a universal form in the bulk of the spectrum: $\langle \rho _{bulk}(e) \rangle = {N b \over 2\pi } \; \sqrt{2 - \left( {b e}\right) ^2}$ with *b* later referred as the bulk spectral parameter and 〈 〉 as the ensemble average; (note here $\langle \rho _e(e) \rangle$ is normalised to *N*: $\int \langle \rho _e(e) \rangle \; \text{d}e =N$). This gives the bulk spectral width as

25 $$\begin{aligned} \omega _c = {2 \sqrt{2} \over b} = {2 N \over \pi \langle \rho _{bulk}(0) \rangle }. \end{aligned}$$

As discussed in detail in Ref.^28^, *b* can be expressed as

26 $$\begin{aligned} b \approx \; { 36 \over \eta \; \sqrt{ z \; g_0} \; A_H} \; {y^6 \over (1+y)^6} = { 9 \over 4 \; \sqrt{ 3 } \; A_H } \; \left( {y \over 1+y}\right) ^{9/2}, \end{aligned}$$

with $A_H$ as the Hamaker constant of the material, *z* as the average number of nearest neighbors of a given molecule, $g_0$ as the number of molecules in the basic block, $\eta ={{\mathcal {N}}}-1$ with ${{\mathcal {N}}}$ as the number of relevant vibrational energy levels in a molecule). Based on the structural stability analysis of the amorphous systems, *z* is predicted to be of the order of 3 (for a three dimensional block)^4–6^. Further ${\mathcal N}$ corresponds to the number of single molecule states participating in dispersion interaction with another molecule. Alternatively, this is the number of dipole transitions among vibrational states of a molecule due to dispersion interaction with another one. Usually the allowed number of such transitions is 3 ($\delta m = 0, \pm 1$ with *m* as the quantum number of the state); in any case weak nature of the dispersion interaction rules out higher number of such transitions).

For comparisons with TTLS model, it is worth noting that ${1 \over \omega _c \Omega _b}$ is of the order of the bulk-density per unit volume. This in turn renders $\langle Q_{a}^{-1} \rangle _{e, \omega }$ given by Eq. (24) analogous to that of TTLS model: $\langle Q_{a}^{-1} \rangle _{e, \omega }^{\text{TTLS}}= {\pi \; \gamma ^2 \; {{\overline{P}}} \over 2 \; \rho _m \; c_a^2 }$ with ${{\overline{P}}}$ as the density of states of TTLS per unit volume.

#### For super block

Equation (24) corresponds to the average coefficient of attenuation in a basic block. Proceeding exactly as above, the average coefficient for a super block, say $\langle Q_{a}^{-1} \rangle ^{sup}_{e, \omega }$, can also be obtained. The steps are as follows. Equation (19) is now replaced by the relation

27 $$\begin{aligned} \sum _{m, n=1}^{N^g} \langle \Gamma _{\alpha \beta ; mn} \; \Gamma _{\gamma \delta ; n m } \rangle _{e} ={ N^g W_c \; \Omega \over \pi } \left( q_c \; \delta _{\alpha \beta } \delta _{\gamma \delta } + \delta _{\alpha \gamma } \delta _{\beta \delta } + \delta _{\alpha \delta } \delta _{\beta \gamma } \right) \; \langle \text{Im} \; \chi _t \rangle ^{sup}_{e.\omega }, \end{aligned}$$

where $\Gamma _{\alpha \beta ; mn}$ refers to the matrix element of $\Gamma _{\alpha \beta }$ in the eigenbasis of *H* (Eq. (10)). But noting that the left side of Eq. (27) can be rewritten as $\langle \text{Tr} (\Gamma _{\alpha \beta ; mn})^2 \rangle$ and is therefore basis-invariant, it can be evaluated in the eigenbasis of $H_0$ i.e the product basis of single block states referred as $|E^0_n\rangle$, $n=1 \rightarrow N^g$. Using

28 $$\begin{aligned} \Gamma _{\alpha \beta ; mn} = \sum _{s=1}^g \Gamma ^{(s)}_{\alpha \beta ; mn}, \end{aligned}$$

along with $\langle \Gamma ^{(s)}_{\alpha \beta ; mn}\Gamma ^{t)}_{\alpha \beta ; mn} \rangle =0$, it is easy to see that

29 $$\begin{aligned} \sum _{m, n=1}^{N^g} \langle \Gamma _{\alpha \beta ; mn} \; \Gamma _{\gamma \delta ; n m } \rangle _{e} = g \; N^{g+1} \; \nu ^2. \end{aligned}$$

The above follows because $\Gamma ^{(s)}_{\alpha \beta ; mn} \not = 0$ only if the product states $|E^0_m\rangle$ and $|E^0_n\rangle$ differ only by the contribution from the $s^{th}$ basic block. Further this also implies that the relevant spectral averaging for the super block is same as that of a basic block i.e $W_c = w_c$. The above, along with the definition $\langle Q_{a}^{-1} \rangle ^{sup}_{e, \omega } = ( \pi \; \rho _m \; c_a^2)^{-1} \; \langle \text{Im} \; \chi _a \rangle ^{sup}_{e.\omega }$ and $\Omega =g \; \Omega _b$, now leads to

30 $$\begin{aligned} \langle Q_{a}^{-1} \rangle ^{sup}_{e, \omega }&= { N \; g \; \nu ^2 \over \omega _c \; \rho _m \; c_a^2 \; \Omega } = { \gamma ^2 \over \omega _c \; \rho _m \; c_a^2 \; \Omega _b }. \end{aligned}$$

A comparison of the above result with Eq. (24) clearly indicates that

31 $$\begin{aligned} \langle Q_{a}^{-1} \rangle ^{sup}_{e, \omega } =\langle Q_{a}^{-1} \rangle _{e, \omega }. \end{aligned}$$

## Qualitative universality of $Q_{a}^{-1}$

Based on unretarded dispersion interaction between molecules, Eq. (24) relates the ultrasonic attenuation coefficient $\langle Q_a^{-1} \rangle$ to the bulk spectrum width $\omega _c$ and thereby bulk spectrum parameter *b*. Equation (26) expresses *b* in terms of the molecular properties. Further as discussed in Ref.^33^ in detail, the size *t* of the basic block can be expressed as

32 $$\begin{aligned} t^2 = { R_0^3\over 4 \; R_v}. \end{aligned}$$

Here $R_v$ is the distance of closest separation between two molecules in the material and $R_0$ is a length scale at which the strength of dispersion interaction between two molecules (i.e. basic structural units^28^) is equal to the phonon mediated coupling of their stress fields^33^

33 $$\begin{aligned} R_0^3 = {\rho _m \; c^2 \; C_6\over 8 \gamma _m^2}, \end{aligned}$$

with $C_6$ as the dispersion coefficient and $\gamma _m$ as the coupling strength of the stress fields of the molecules. Using $\Omega _b = s \; t^3$, the above then gives the volume $\Omega _b$ of the basic block in terms of molecular parameters. Further, as discussed in Ref.^33^, the number of molecules in a basic block can be given as

34 $$\begin{aligned} g_0 = {\Omega _b \over \Omega _{\text{eff}}} \approx {1 \over (1+y)^3} \; \left( {t \over R_m}\right) ^3 = {y^3 \over 8 \; (1+y)^3} \; \left( {R_0 \over R_v}\right) ^{9/2}, \end{aligned}$$

with $y={R_v \over R_m}$ where $R_m$ is the radius of the molecule.

A combination of the above relations then gives $\langle Q_a^{-1} \rangle$ in terms of the molecular properties. This can be derived as follows. A substitution of Eq. (26) in Eq. (24), along with above relations for $t, R_0$ and $g_0$ and $s=4 \pi /3$, leads to

35 $$\begin{aligned} \langle Q_{a}^{-1} \rangle _{e, \omega }&\approx { 64 \; \gamma ^2 \over 2 \; s \; \eta \sqrt{2 z g_0 } \; \rho _m \; c^2 \; C_6} \; {R_v^6 \over t_0^3 }, \end{aligned}$$

where, as discussed in Sect. I of SI files, $\gamma$, the coupling strength of basic blocks can be expressed in terms of that of molecules i.e $\gamma _m$,

36 $$\begin{aligned} \gamma ^2 \approx {4 \; \pi \; g_0 \over K \sqrt{2}} \; \gamma _m^2, \end{aligned}$$

with

37 $$\begin{aligned} K^2=18 \left( 5-4 \; {c_t^2 \over c_l^2}\right) . \end{aligned}$$

Using Eq. (33) to replace $C_6$ in the above equation leads to

38 $$\begin{aligned} \langle Q_{a}^{-1} \rangle _{e, \omega }&\approx { 8 \; \pi \; \sqrt{g_0} \over s \; \eta \; \sqrt{z} \; K} \; {R_v^6\over R_0^3 \; t^3}, \end{aligned}$$

39 $$\begin{aligned}&= { 32 \; \pi \; \over s \; \eta \; \sqrt{2 z} \; K} \; \left( {y\over (1+y)}\right) ^{3/2} \; \left( {R_v\over R_0}\right) ^{21/4}. \end{aligned}$$

Here the 2nd equality is obtained by substitution of *t* and $g_0$ from Eqs. (32) and (34). Further, as mentioned below Eq. (26), $\eta =2$ (with ${{\mathcal {N}}}=3$ as the number of allowed dipole transitions in a molecule) and *z* as the number of nearest neighbors of a molecule (those only interacting by VWD). The quantitative information about $R_v$ available for a wide range of materials suggests $R_v \sim R_m$^37^. Taking $y= {R_v \over R_m} \sim 1$ leads to, from Eq. (34), $g_0 \approx 8$. Assuming uniform mass density, this also implies only three nearest neighbor molecules to a given molecule within a spherical basic block of radius $t=\sqrt{ R_0^3\over 4 \; R_v}$ and therefore $z=3$.

Following from Eq. (39), an almost quantitative universality of $Q^{-1}$, as experimentally predicted for amorphous systems^1^, is not directly obvious. This however follows by noting that the length scales $R_0$ and $R_v$ are related by a constant: $R_0=4 R_v$^33^. Further the ratio ${c_t \over c_l}$, and therefore *K* (from Eq. (37)), is known to be almost constant for many structural glasses^24,35^. Substitution of ${R_0\over R_v} =4$ in Eq. (39) along with $y \approx 1$ and $s=4 \pi /3$ leads to an almost material independent value of average internal friction

40 $$\begin{aligned} \langle Q_{a}^{-1} \rangle _{e, \omega } \approx 2.83 \times 10^{-4} \times \left( 1.25 - {c_t^2 \over c_l^2}\right) ^{-1/2}. \end{aligned}$$

Previous experiments indicate that ${c_l \over c_t}$ varies between $1.5 \rightarrow 2$, thus changing $\langle Q_{a}^{-1} \rangle _{e, \omega }$ within $10\%$ only.

Further insight in the above result can be gained by rewriting $\langle Q_{a}^{-1} \rangle _{e, \omega }$ in terms of $g_0$, the number of molecules in a basic block. Substitution of Eq. (34) in Eq. (39) gives $\langle Q_{a}^{-1} \rangle _{e, \omega } \propto {g_0^{-7/6} }$. Further, using the relation $R_0=4 R_v$^33^, Eq. (34) gives a constant, system-independent number of the molecules within each block: $g_0 ={64 \; y^3 \over (1+y)^3}$. This in turn leads to a material independent value of the average ultrasonic attenuation coefficient $\langle Q^{-1}\rangle$. The above along with the definition given in Eq. (12) further suggests that the universality is brought about by the phonons of wavelength $\lambda \sim g_0 \; l$ with *l* as their mean free path.

Taking typical value $R_m \sim 2.5{-}3.5 \; \AA$ gives $R_0 \sim 10{-}15 \; \AA$ which corresponds to the length scale for medium range topological order (MRO) ($10 \; \AA \rightarrow 30 \; \AA$) (also see Table 3 of Ref.^33^ for glass specific values of $R_0$) . This is as expected because VWD interactions are negligible beyond MRO and other interactions start dominating beyond this length scale.

Equation (40) is the central result of this paper. As described above, it is based on a balancing of the VW forces with phonon induced interactions among molecules at MRO length scales in amorphous systems. The universal aspects of this competition, as described above, then result in the qualitative universality of $\langle Q_{a}^{-1} \rangle _{e, \omega }$ which is consistent with experimental observations^1^. Note, based on the type of the experiment, the observed data for a glass often vary from one experiment to another (see for example, the values of tunnelling strengths $C_{l,t}$ in Ref.^1,35^).

## Comparison with experimental data

Equations (39) and (40) both give theoretical formulations for the internal friction in terms of the molecular properties. Eq. (40) however is based on an additional prediction $R_0= 4 R_v$, derived and analyzed in Ref.^33^. This motivates us to compare both predictions, namely, Eqs. (39) and (40), with experimental data for 18 glasses given by two different studies^1^ and^35^.

A comparison of Eq. (40) with experiments requires the information only about $c_l, c_t$ and is straightforward. But Eq. (39) depends on many other material properties and needs to be rewritten as follows. As discussed in Ref.^33^, $R_0$ can be expressed in terms of molecular properties

41 $$\begin{aligned} R_0^3 = {(1+y)^6 \; c^2 \; A_H \; M \; \Omega _m \over 8 \; \pi ^2 \; \gamma _m^2 \; N_{av}}. = {(1+y)^6 \; \; A_H \; M^2 \; c^2 \over 8 \; \pi ^2 \; N_{av}^2 \; \gamma _m^2 \; \rho _m}. \end{aligned}$$

Substitution of the relation $\Omega _m = {4 \over 3} \pi \; R_m^3$ in Eq. (41) gives

42 $$\begin{aligned} \left( R_0\over R_v \right) ^3 = {1\over y^3} \; \left( R_0\over R_m \right) ^3&= {(1+y)^6 \over y^3} \; { M \; A_H \over 6 \; \pi \; N_{av} } \; \left( {c \over \gamma _m} \right) ^2. \end{aligned}$$

Here *c*, as the speed of sound, and $\gamma _m$, as the phonon mediated coupling constant between molecules, have directional dependence: $c=c_l,c_t$ and $\gamma _m=\gamma _l, \gamma _t$ with subscripts *l*, *t* referring to longitudinal and transverse direction, respectively. The above along with Eq. (39) gives,

43 $$\begin{aligned} \langle Q_{a}^{-1} \rangle _{e, \omega }&= {48 \; f(y) \over \eta \; \sqrt{2 \; z} \; K} \; \left( {6 \; \pi \; N_{av} \over M \; A_H} {\gamma _a^2 \over c_a^2} \right) ^{7/4}, \end{aligned}$$

where $f(y)={y^{27/4} \over (1+y)^{12}}$ with $\eta =2$, $z=3$ and the subscript $a=l,t$. For later reference, note *f*(*y*) is almost same for $y=1$ and $y=1.5$: $f(1)=2.44 \times 10^{-4}$ and $f(1.5) =2.59 \times 10^{-4}$.

As standard TTLS model is a special case of our generic block model, the available information for the coupling constants in the former case can be used for the latter (Note TTLS model is based on the presence of some two level atoms/molecules (TLS) as defects. The coupling constants of the molecules within a block due to molecule–phonon interaction can then be taken same as those of TLS). The TLS coupling constants are related to tunnelling strength $C_{a}$, defined as

44 $$\begin{aligned} C_{a} = {{{\overline{P}} } \; \over \rho _m} \; \left( {\gamma _a \over c_a}\right) ^2, \end{aligned}$$

with ${{\overline{P}} }$ as the spectral density of tunnelling states. According to tunnelling model,

45 $$\begin{aligned} C_{a} = {2\over \pi } \; \langle Q_{a, pohl}^{-1} \rangle . \end{aligned}$$

As the experimental results are usually given in terms of TTLS parameters $C_l, C_t$, we define the analog of $C_a$ for our case for comparison

46 $$\begin{aligned} {{\mathcal {B}}_a} = {2\over \pi } \; \langle Q_{a,pohl}^{-1} \rangle = 2 \; \langle Q_a^{-1} \rangle . \end{aligned}$$

The above along with Eqs. (43) and (44) then gives

47 $$\begin{aligned} {{\mathcal {B}}}_a&= {6 \; f(y) \over \eta \; \sqrt{z} \; K} \; \left( {6 \; \pi \; N_{av} \over M \; A_H} {\rho _m \; C_a \over { {\overline{P}}}}\right) ^{7/4}. \end{aligned}$$

### Determination of physical parameters

Both definitions in Eqs. (45) and (46) refer to same physical property, i.e., internal friction, thus implying ${\mathcal B}_a = C_a$. From Eq. (47), however, ${{\mathcal {B}}_a}$ depends on many other parameters besides $C_a$ which vary from one glass to another. Although, not obvious a priori how the two can be equal, this is indeed necessary if our theoretical prediction in Eq. (47) is consistent with the experimental values for $\langle Q_a^{-1}\rangle$. To verify the equality, we pursue a detailed quantitative analysis of ${{\mathcal {B}}}_l, {{\mathcal {B}}}_t$ for 18 glass. The required values of $c_l, c_t$ to determine *K* along with $\rho _m$ and $\overline{P}$ are taken from^35^. The information about $C_a, A_H$ and *M* for the purpose is obtained as follows.

(i) $C_l, C_t$: Using ultrasonic absorption data, the study^35^ determines $C_l, C_t$ as adjustable parameters for 18 glasses; these values are displayed in columns 4 and 10 of Table 1 (referred as $C_{l,bm}$ and $C_{t,bm}$). The corresponding results for ${{\mathcal {B}}}_{l, t}$, derived from Eq. (47), are displayed in columns 3 and 9 of Table 1 (with notations defined in Table captions). The $C_l, C_t$-values mentioned in Ref.^1^ for some of the glasses are different from^35^ (although $c_l, c_t$ values are same in both studies) which then lead to, from Eq. (44), different values for $\gamma _l, \gamma _t$. Further note that the study^1^ considers data from two different experimental approaches, namely, acoustic and flexural) and the results for $C_l, C_t$ values vary from one experiment to another. This motivates us to compare Eq. (47) with two sets of data given in Ref.^1^ too. The $C_l, C_t$ values from^1^ are displayed in Table 1 in columns 6, 8, 12, 14; the latter along with $\rho _m$ and ${{\overline{P}}}$ (both given in Table 2) are used to obtain corresponding theoretical predictions for ${{\mathcal {B}}}_l, {{\mathcal {B}}}_t$, given in columns 5, 7, 11, 13.
(ii) *M*: As Eq. (47) depends on $M^{7/4}$, a correct estimation of *M* is important too. Two options available to determine *M* are (i) mass of the basic structural unit which dominates the structure of the glass and participates in the dispersion interaction (later referred as vwd unit), or, (ii) the molecular mass of the glass (later referred as formula unit); (here, for example for $SiO_2$ glass, $SiO_2$ is the formula unit but dominant structural unit can be $SiO_4$ or *Si*(*SiO*4)). Clearly, with dispersion interaction as the basis of our analysis, it is reasonable to use the 1st option . To analyze the influence however we consider both options to calculate ${{\mathcal {B}}}_l, {{\mathcal {B}}}_t$. The details of dominant structural unit for each glass and its mass, referred as $M_1$, is discussed in Sect. II of SI files. The formula mass, labelled here as $M_2$, corresponds to weighted summation of the molar masses of each constituent of the glass: for the latter consisting of *n* components $X_k$, $k=1 \rightarrow n$, with their molar mass as $m_k$ and weight percentage as $p_k$, $M_2= \sum _{k=1}^n p_k \; m_k$. The glass composition for the 18 glasses is given in Sect. II of SI files and their $M_1, M_2$ values are displayed in Table 2.
(iii) $A_H$: for materials in which spectral optical properties are not available, two refractive-index based approximation for $A_H$ namely, standard Tabor–Winterton approximation (TWA) (appropriate for low refractive index materials, $n < 1.8$) and single oscillator approximation (SOA) (for higher indexes $n > 1.8$), provide useful estimates^38^ . The $A_H$ for 18 glasses listed in Table 2 are based on these approximations (with details given in Ref.^28^).

**Table 1:** Comparison of theoretical and experimental values of internal friction for 18 glasses with $M=M_1$: Here the theoretcial result from Eq. (47) labelled as ${{\mathcal {B}}}_{a,xx}$, with $a \equiv l, t$ are displayed in odd numbered columns for $M=M_1$.

Index	Glass	${{\mathcal {B}}}_{l,bm}$	$C_{l,bm}$	${{\mathcal {B}}}_{l,p1}$	$C_{l,p1}$	${{\mathcal {B}}}_{l,p2}$	$C_{l,p2}$	${{\mathcal {B}}}_{t,bm}$	$C_{t,bm}$	${{\mathcal {B}}}_{t,p1}$	$C_{t,p1}$	${{\mathcal {B}}}_{t,p2}$	$C_{t,p2}$	${{\mathcal {B}}}_{th}$
Units		$\times 10^4$	$\times 10^4$	$\times 10^4$	$\times 10^4$	$\times 10^4$	$\times 10^4$	$\times 10^4$	$\times 10^4$	$\times 10^4$	$\times 10^4$	$\times 10^4$	$\times 10^4$	$\times 10^4$
1	a-SiO2	4.50	3.10	4.51	3.00	4.00	2.80	3.81	2.90	4.51	3.00	4.78	3.10	3.11
2	BK7	3.09	2.70					4.38	3.30					3.01
3	As2S3	0.76	1.60	1.64	2.30	0.69	1.40	1.48	2.00	0.96	1.70			2.88
4	LASF7	1.92	1.20	4.81	2.00			1.84	1.16					3.07
5	SF4	2.58	2.20					3.89	2.80					2.97
6	SF59	4.56	2.30					6.38	2.80					2.95
7	V52	2.46	4.00	5.03	6.00			3.46	4.90	4.18	5.40			2.88
8	BALNA	1.82	3.80					2.71	4.80					2.87
9	LAT	2.29	3.80					2.15	3.70					2.96
10	a-Se	0.65	1.20	0.88	2.20			0.82	2.20	1.42	2.90			2.86
11	Se75Ge25								0.90					
12	Se60Ge40	1.86		1.83	1.30			0.14	0.30					2.99
13	LiCl:7H2O	3.44	7.20	3.29	7.00			7.67	11.36	6.14	10.0			2.82
14	Zn-Glass	2.09	3.00					2.79	3.60					2.82
15	PMMA	1.55	2.00	4.57	3.70	3.35	3.10	4.90	3.70	7.21	4.80	9.73	5.70	2.82
16	PS	2.44	3.60			11.13	8.30	4.79	5.00	16.52	10.40	9.99	7.80	2.87
17	PC	1.00	1.80	3.51	3.50			3.19	3.30	31.23	12.20	20.16	9.50	2.77
18	ET1000	2.06	2.80	5.96	5.00			Inf						2.52

The 2nd subscript *xx* refers to the particular experiment used to obtain required parameters in Eq. (47): $xx \equiv bm,p1, p2$ for data from^35^, $xx \equiv p1$ for accoustic data from^1^, $xx \equiv p2$ for flexural data from^1^). The values used for $M_1, c_l, c_t$ to obtain ${{\mathcal {B}}}_{a,xx}$ are given in Table 2, with experimental data for $C_a$ given in adjacent even-numbered columns. The last column gives our theoretical prediction from Eq. (40).

**Table 2:** Physical parameters for 18 glasses.

Index	Glass	$\rho _m$	$c_l$	$c_t$	$\gamma _l$	$\gamma _t$	${{\overline{P}}}$	$A_H$	$M_1$	Vwd unit	$M_2$
		$\times 10^3 \,{\text{{kg}}/\text{{m}}^3}$	km/s	km/s	ev	ev	$10^{45}/\text{{J m}} ^3$	$\times 10^{-20} \; \text{{J}}$	g/mole		g/mole
1	a-SiO2	2.20	5.80	3.80	1.04	0.65	0.8	6.31	120.09	[$Si(SiO_4$]	60.08
2	BK7	2.51	6.20	3.80	0.9	0.65	1.1	7.40	92.81	[$SiO_4$]	65.84
3	As2S3	3.20	2.70	1.46	0.26	0.17	2.0	19.07	32.10	[*S*]	246.03
4	LASF	5.79	5.64	3.60	1.46	0.92	0.4	12.65	167.95	[*LASF*]	221.30
5	SF4	4.78	3.78	2.24	0.72	0.48	1.1	8.40	136.17	[$Si_2O_5$]	116.78
6	SF59	6.26	3.32	1.92	0.77	0.49	1.0	14.05	92.81	[$SiO_4$]	158.34
7	V52	4.80	4.15	2.25	0.87	0.52	1.7	8.37	167.21	[$ZrF_4$]	182.28
8	BALNA	4.28	4.30	2.30	0.75	0.45	2.1	6.87	167.21	[$ZrF_4$]	140.79
9	LAT	5.25	4.78	2.80	1.13	0.65	1.4	9.16	205.21	[$ZrF_6$]	215.69
10	a-Se	4.30	2.00	1.05	0.25	0.14	2.0	18.23	78.96	[*Se*]	78.96
11	Se75Ge25	4.35	0.00	1.24		0.15	1.0	22.19	77.38	[$Se_3Ge_1$]	77.38
12	Se60Ge40	4.25	2.40*	1.44*		0.16	0.4	23.56	76.43	[$Se_3Ge_1$]	76.43
13	LiCl:7H2O	1.20	4.00	2.00*	0.62	0.39	1.4	4.75	131.32	[$Li(H_2O)Cl_3$]	168.50
14	Zn-Glass	4.24	4.60	2.30	0.70	0.38	2.2	7.71	103.41	[$ZnF_2$]	103.41
15	PMMA	1.18	3.15	1.57	0.39	0.27	0.6	6.10	102.78	[*PMMA*]	102.78
16	PS	1.05	2.80	1.50	0.20	0.13	2.8	6.03	27.00	[$CH-CH2$]	105.15
17	PC	1.20	2.97	1.37*	0.28	0.18	0.9	6.00	77.10	[$C_6H_5$]	252.24
18	ET1000	1.20	3.25		0.35	0.22	1.1	4.91	77.10	[$C_6H_5$]	77.10

The table lists the available data for the physical parameters appearing in Eqs. (39), (43) and (47). The $\rho , c_l, c_t, {{\overline{P}}}$ data from^35^ (or^1^ if not available in Ref.^35^) is displayed in columns 3rd, 4th, 5th and 8th, respectively. The columns 6th and 7th give the $\gamma _l$ and the $\gamma _t$ values, taken from Ref.^35^ except for few cases; for those marked by a star (*), the values are obtained either from^1^ or from $C_l, C_t$ values given in Ref.^35^ along with Eq. (45). Although not used for our analysis, the $\gamma$ values are included here for completeness). The $A_H$ values given in columns 9th are taken from Ref.^28^. The molar mass values, referred as $M_1$ for the vwd unit along with its composition is given in columns 10th and 11th and the mass $M_2$ for formula unit (same as glass molecular weight) in column 12th respectively.

**Table 3:** Comparison of theoretical and experimental values of internal friction for 18 glasses with $M=M_2$: All other details here are same as in Table 1.

Index	Glass	${{\mathcal {B}}}_{l,bm}$	$C_{l,bm}$	${{\mathcal {B}}}_{l,p1}$	$C_{l,p1}$	${{\mathcal {B}}}_{l,p2}$	$C_{l,p2}$	${{\mathcal {B}}}_{t1,bm}$	$C_{t,bm}$	${{\mathcal {B}}}_{t,p1}$	$C_{t,p1}$	${{\mathcal {B}}}_{t,p2}$	$C_{t,p2}$	${{\mathcal {B}}}_{th}$
Units		$\times 10^4$	$\times 10^4$	$\times 10^4$	$\times 10^4$	$\times 10^4$	$\times 10^4$	$\times 10^4$	$\times 10^4$	$\times 10^4$	$\times 10^4$	$\times 10^4$	$\times 10^4$	$\times 10^4$
1	a-SiO2	15.11	3.10	15.17	3.00	13.44	2.80	12.81	2.90	15.17	3.00	16.06	3.10	3.11
2	BK7	5.64	2.70					8.00	3.30					3.01
3	As2S3	0.02	1.60	0.05	2.30	0.02	1.40	0.04	2.00	0.03	1.70			2.88
4	LASF7	1.19	1.20	2.97	2.00			1.14	1.16					3.07
5	SF4	3.37	2.20					5.09	2.80					2.97
6	SF59	1.79	2.30					2.50	2.80					2.95
7	V52	2.11	4.00	4.33	6.00			2.97	4.90	3.60	5.40			2.88
8	BALNA	2.45	3.80					3.67	4.80					2.87
9	LAT	2.10	3.80					1.97	3.70					2.96
10	a-Se	0.65	1.20	0.88	2.20			0.82	2.20	1.42	2.90			2.86
11	Se75Ge25								0.90					
12	Se60Ge40	1.86		1.83	1.30			0.14	0.30					2.99
13	LiCl:7H2O	2.22	7.20	2.13	7.00			4.96	11.36	3.97	10.0			2.82
14	Zn-Glass	1.35	3.00					1.80	3.60					2.77
15	PMMA	1.55	2.00	4.57	3.70	3.35	3.10	4.90	3.70	7.21	4.80	9.73	5.70.	2.82
16	PS	0.23	3.60			1.03	8.30	0.44	5.00	1.53	10.40	0.93	7.80	2.87
17	PC	0.13	1.80	0.44	3.50			0.40	3.30	3.92	12.20	2.53	9.50	2.77
18	ET1000	2.06	2.80	5.96	5.00									2.52

### Quantitative analysis

As mentioned above, Eq. (40) for $\langle Q^{-1}_{a}\rangle$ is based on relation $R_0=4 R_v$ but Eq. (47) is based only on Eq. (33); (note Eq. (47) follows from Eq. (43)). The present analysis therefore provides two pathways to theoretically determine $\langle Q^{-1}_{a}\rangle$, one based on constant ratio of two short range length scales and other on molecular properties. The first pathway requires the information about $c_l, c_t$ only but the second one also requires a prior information about the tunnelling strength $C_a$. The reported experimental data for the latter however varies significantly from one experiment to another (as indicated by the data from Refs.^1,35^ in even numbered columns of Tables 1, 3). This in turn leads to different values of ${{\mathcal {B}}}_a$ (from Eq. (47)); the latter are displayed in odd-numbered columns of Tables 1 and 3 (for $M_1$ and $M_2$ respectively). Note, as displayed in Table 2, $M_1$ and $M_2$ do not differ significantly for the glass-ceramics and, consequently, the predictions for ${{\mathcal {B}}}_a$ for the two cases are close. However, for single component glasses e.g. SiO2 or where one component dominates (e.g. in BK7), ${{\mathcal {B}}}_l, {{\mathcal {B}}}_t$ predictions based on $M_1$ are closer to experimental data (see Table 1). This in turn provides further credence to the relevance of VW forces in present context.

**Figure 1:**
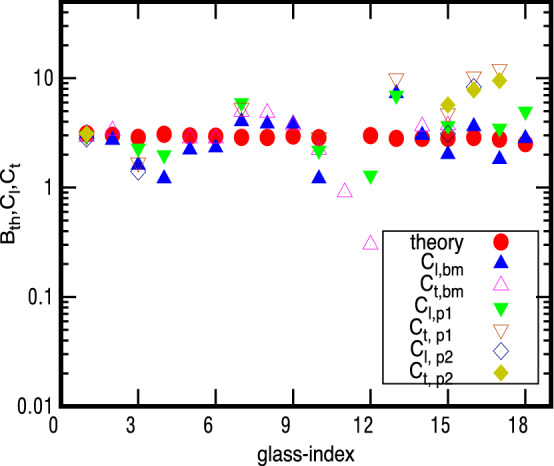
${{\mathcal {B}}}_{th}$- values for 18 glasses: The figure depicts the theoretically predicted ${{\mathcal {B}}}_{th}$ from Eq. (40) and corresponding experimentally known tunneling strengths $C_{a}$ with respect to glass-index (given in 1st column of Table 2). The symbol $C_{a,bm}$ refers to experimental data for tunneling strength from^35^ and $C_{a,p1}$, $C_{a,p2}$ to acoustic and flexural data, respectively, from^1^. The values for $B_{th}$ are also given in the last column of Tables 1 and 3; note these are same for both $M_1, M_2$.

**Figure 2:**
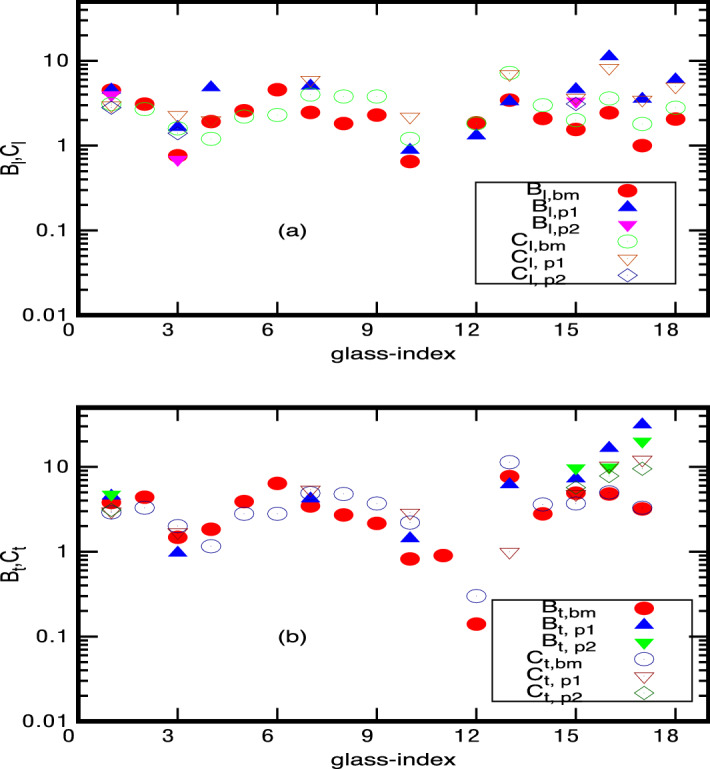
${{\mathcal {B}}}_{a}$-values for 18 glasses (with $M=M_1$): The figure depicts the theoretically predicted ${\mathcal B}_{a}$ and corresponding experimentally known tunneling strengths $C_{a}$ with respect to glass-index (all listed in Table 1). Here ${{\mathcal {B}}}_{a,xx}$ refers to Eq. (47) using tunneling parameters from different experiments (with $xx=bm$ referring to experimental data from^35^, $xx = p1$ to acoustic and $xx=p2$ to flexural data from^1^). The symbols $C_{a,xx}$ refer to experimental data from^35^ and^1^ accordingly.

**Figure 3:**
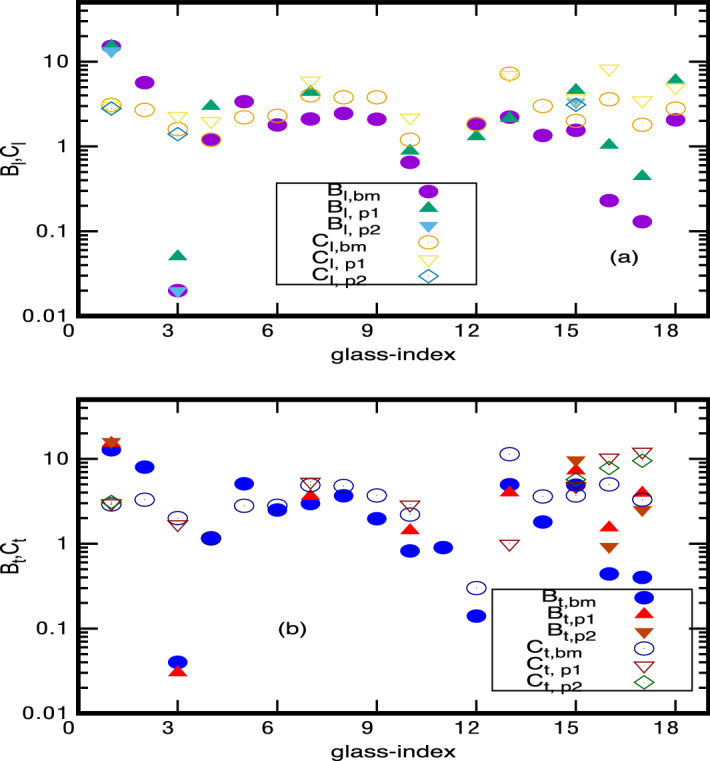
${{\mathcal {B}}}_{a}$-values for 18 glasses (for $M=M_2$): All details are same as in Fig. 2 except that now the results for ${{\mathcal {B}}}_{a,xx}$ from Eq. (47) correspond to $M=M_2$. Note although the correspondence with experiments here is not as good as for $M_1$, the deviation however is still within a factor of 10. As reported in Ref.^1^, the deviation of different experimental results lies also within that range.

**Figure 4:**
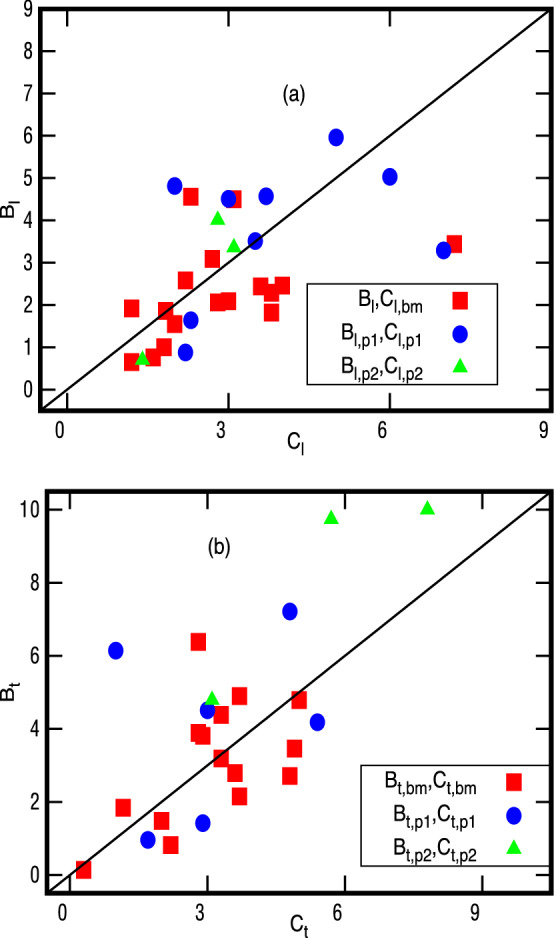
Comparison of ${{\mathcal {B}}}_a$-**values** ($a=l,t$), for 18 glasses from Eq. (47), for $M=M_1$, with their experimentally known tunneling strengths : here the ${\mathcal B}_{a1,xx}$-values correspond to *y*-coordinates of the points marked on the figure and $C_{a,xx}$ to their *x*-coordinates; the details of the labels are same as in Fig. 2. Here the solid line is shown only for visual guidance.

**Figure 5:**
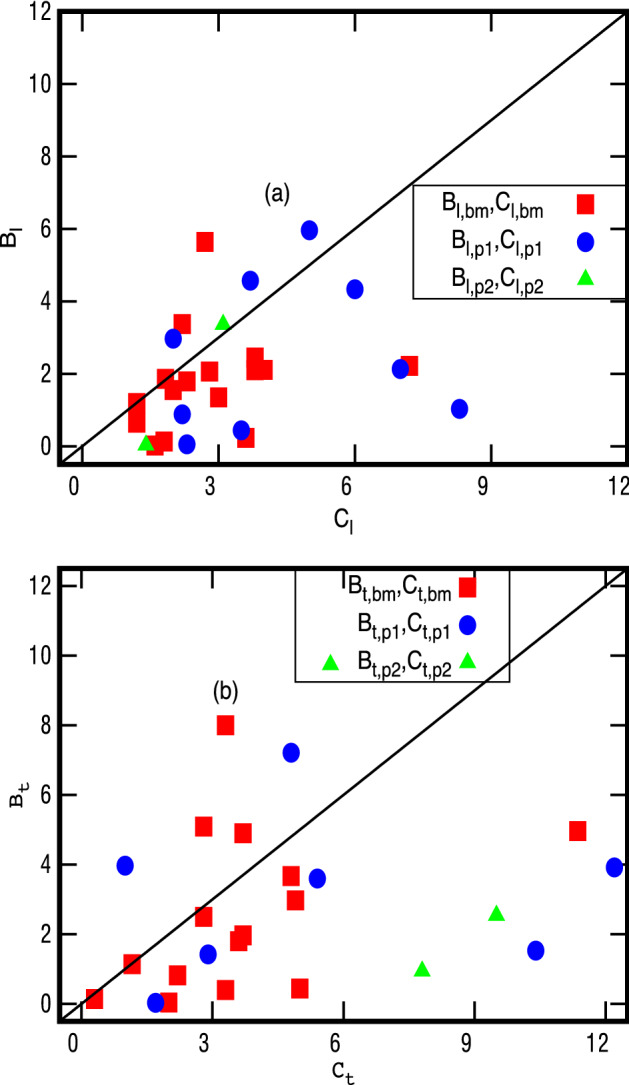
Comparison of ${{\mathcal {B}}}_a$-values ($a=l,t$), for 18 glasses from Eq. (47), for $M=M_2$, with their experimentally known tunneling strengths : here the ${\mathcal B}_{a1,xx}$-values correspond to *y*-coordinates of the points marked on the figure and $C_{a,xx}$ to their *x*-coordinates; the details of the labels are same as in Fig. 2. Here again the solid line is shown only for visual guidance.

The values of ${{\mathcal {B}}}_{th}=2 \langle Q_a^{-1} \rangle$ from Eq. (40), along with corresponding experimental $C_a$ data for each glass, is also illustrated in Fig. 1. The similar comparison based on Eq. (47) is displayed in Fig. 2 for $M=M_1$ and Fig. 3 for $M=M_2$. A direct comparison of theoretical and experimental data is also displayed in an alternative way in Fig. 4 for $M_1$ and in Fig. 5 for $M_2$. As mentioned above, the results for a glass vary from one experiment to other often within a factor of 2 but sometimes more e.g. for polymers (see odd numbered columns of Tables 1 and 3 and also^1^). But the deviation of our theoretical prediction from experiments is usually less than a factor of 2.

Further, a comparison of Figs. 2 and 3 (or Figs. 4, 5) indicates that the results for $M=M_1$ are closer to experimental data, thus indicating the molecules interacting by VWD interaction as an appropriate choice for the present analysis. This is also consistent with our theoretical approach assuming VWD interactions as the relevant interaction for length scales less than MRO.

An important point to note here is that the ${\mathcal B}_{a}$-dependence in Eq. (47) on glass-properties is based only on the product $M . A_{H}$. (This can be seen by substituting $R_0 = 4 R_v$ in Eq. (41) which then gives the ratio ${\gamma _m\over c}$ in terms of $M. A_H$ and thereby leads to an important result ${\gamma _l\over \gamma _t} ={c_l \over c_t}$^33^). The quantitative universality of $\langle Q^{-1}\rangle$ therefore seems to be a reconfirmation of already known relation between $A_H$ and molar volume^39^.

## Discussion

The definition in Eq. (12), along with an almost constant $Q^{-1}_a$, implies a linear relation between the phonon mean free path *l* and its wavelength $\lambda$: $l \sim 10^{3} \lambda$. Within TTLS model, this behavior was explained by two different mechanisms: the low frequency phonons were postulated to be attenuated mainly by a relaxation of TLS defects but high frequency phonons that carry the heat were believed to be resonantly scattered^2,37^. Later on TLS were generalized to soft local atomic potentials (quasi-harmonic oscillators) and their interactions with phonons was attributed to be the cause of a constant $Q^{-1}$^11^. The approach however gave $C_a \sim 1$ i.e., a value three orders of magnitude too large; this later on led to suggestions that only a small fraction of the quasi-harmonic oscillators act as tunneling defects^11,40^.

Although as discussed in Ref.^1^, TTLS model shows good agreement for many glasses, the physical nature of tunnelling entities its not yet fully understood. Further the resemblance of the low-energy excitations in many disordered crystals to those found in amorphous solids strongly suggests their origin not related to long-range order in materials. It is therefore necessary to seek alternative theories especially those based on MRO i.e a length scale dominated by VW forces, present in all materials. This is indeed the case in our approach based only on two scales $R_0$ and $R_v$, the first of the order of MRO and second that of SRO. Note ideas suggesting a role of MRO scales in origin of glass anomalies have appeared in past too e.g.^29,25,32,34^. However these were based on experimentally/ numerically observed existence of structural correlations at these scales and did not explicitly consider the role of molecular interactions.

As Eq. (43) indicates, $Q^{-1}$ depends only on the ratio $R_0 \over R_v$ which in turn is related to $g_0$, the number of molecules within the block. As the molecules interact by VW forces e.g by formation of induced dipoles that decay rapidly (i.e $r^{-6}$) with *r* as the distance between molecules, the dominant contribution comes from the nearest neighbor molecules only. Under acoustic perturbation, the molecules go to vibrational excited state by absorbing the energy from sound waves which triggers the induced dipole interactions among neighboring molecules. As this number can not vary much from one glass to another (assuming three dimensional structure) except for thin films, this results in a constant value of $Q^{-1}$. This also explains observed deviation in some thin films (see^1^). As indicated in Table 1, the value of $Q^{-1}$ given by our approach for 18 glasses is in good agreement with experimental data.

Further physical insight in this consistency can be given as follows. As discussed in detail in Ref.^33^, $R_0$ is also the size of the basic block and can be expressed in terms of molecular parameters. At large $\lambda > 2 R_0$, the basic block subunits within a macroscopic glass block respond as an array of periodic structures which in turn ensures large mean free paths, thereby reducing the attenuation. For $\lambda \le 2 R_0$ however the orientational disorder of the induced dipoles at MRO scale or less affects the phonon dynamics causing their scattering and thereby localization. Thus $R_0$ is a relevant scale for the sound absorption and thereby attenuation in glasses; as discussed in Ref.^33^, our $R_0$ is approximately the same as *R* of^32^ (see Table 1 of Ref.^32^). The 2nd scale $R_v$ appears in the wave-dynamics due to its sensitivity to the number of interacting molecules (from Eq. (34)). As the change of phonon dynamics occurs at length scale $R_0$, the Ioffe-Regel (IR) frequency $\omega _{ir}$ is therefore expected to correspond to $c_a/2R_0$, marking the transition from the well-defined acoustic like excitations to those characteristic of basic block, with $c_a=c_l,c_t$ as the sound velocity in the medium^33^. A comparison of our theoretical prediction $\omega _{ir}=c_a/2R_0$ with experimental available boson peak frequencies further indicates their closeness.

At this stage, it is worth reviewing the main assumptions made to arrive at our theoretical predictions:

(i) The interactions within the block are assumed to be homogeneous. The assumption was used in “Ultrasonic attenuation coefficient: relation with stress matrix” section for the random matrix modelling of the Hamiltonian as well as in linear response theory for $Q^{-1}$. This puts an upper limit on the allowed block size. As discussed in Ref.^33^, the size of the block turns out to be of the medium range order $\sim 3\, \text{nm}$ with only 8 molecules within, the assumption of homogeneity can be well satisfied.
  Any block of bigger size would include both dispersion as well as phonon-coupling among molecules and thereby lead to inhomogeneity of the interactions. The theory in principle can still be adapted to analyze a super block consisting of bigger basic block sizes (as in Ref.^23^) but it would need many modifications including the use of sparse random matrices. (Note with a radius $R_0$, the basic block considered here satisfies this condition).
(ii) The blocks are assumed to be of spherical shapes. This is a natural choice, keeping in view especially of the spherical shape of the molecules (although the latter is also an assumption but a standard one). It also helps a simpler technical formulation of the derivations. Alternatively, basic blocks of arbitrary shape can also be chosen but that is at the cost of technical complexity of intermediate steps of the derivation. We believe that although the ratio ${R_0 \over R_m}$ may vary slightly with shape but it will be compensated by the structure parameter *s*, thus leaving theoretical prediction in Eqs. (43) and (47) almost unaffected.
(iii) The interaction between phonon and non-phonon degrees of freedom are assumed to be weak, allowing linear response of the blocks to external perturbation.
  The phonon mediated perturbation is assumed to access all *N* levels of the basic block Hamiltonian ($N = {{\mathcal {N}}}^g =3^g$) within spectral range $\omega _c \sim 10^{-18} \; \text{{J}}$ (from Eq. (25). Although this gives the mean energy level spacing in the spectral bulk as $\Delta _b \approx {\omega _c \over N}$ for a basic block is $\sim 10^{-22} \; \text{J}$, the mean level spacing in the lower edge of the spectrum however is much smaller and levels can be accessed by thermal perturbation at low temperatures $T \sim 1\, \text{K}$.
(iv) The dominant interactions at at MRO length scales of the glasses are non-retarded dispersion forces among molecules. This is applicable only to insulator glasses and needs to be replaced for other cases.
(v) The theoretical results presented here (Figs. 1, 2, 3, 4, Tables 1, 2, 3) are obtained from Eq. (47) with $y=R_v/ R_m \sim 1$ for the molecules interacting by VWD. In general *y* fluctuates from one glass to another with 1 as its average value; the glass-specific values for *y* should be taken, in principle, for better accuracy. However as noted below Eq. (43), *f*(*y*) remains almost same for $y=1$ and $y=1.5$: $f(1)=2.44 \times 10^{-4}$ and $f(1.5) =2.59 \times 10^{-4}$. The fluctuation of *y* therefore does not seem to have significant effect of our results.
(vi) The ${{\mathcal {B}}}_{l}, {{\mathcal {B}}}_{t}$ values given in Table 1 are obtained by approximate $A_H$ values used in Eq. (43); we believe the results could be improved if exact values of $A_H$ are used (see^39,38^). Further our results given in Table 1 are based on the Hamaker constant of the molecules interacting in vaccum. The vwd unit is however the dominant cation surrounded by other molecules; the interaction between two cations is therefore mediated by other molecules. It is natural to query, therefore, how the ${{\mathcal {B}}}_a$ results will be affected if $A_H$ values in the relevant medium are considered.

## Conclusion

In the end, we summarize with our main ideas and results.

Based on experimental evidence of ordered structure in glasses below MRO ($10 \rightarrow 30\, \AA$) and its lack above, we describe a macroscopic size glass material as elastically coupled, spherical shape, generic blocks, with homogeneous dispersion interaction within each such block. A random matrix modelling of their hamiltonian and linear response to an external strain field, then relates the low temperature averaged ultrasonic attenuation coefficient for the glass to a ratio of molecular length scales and a ratio of longitudinal and transverse sound speeds in the amorphous solid; the theoretical justification supported by numerical evidence for the former and experimental one for the latter indicate these ratios to be almost material independent. This in turn reveals the qualitative universality of the coefficient which is consistent with experimental observations in the temperature regime $1 \; K \rightarrow 10\, \text{{K}}$^1^.

The central result of our work is given by Eqs. (39) and (40) with main assumptions summarised in “Conclusion” section. An important insight revealed by our formulation is the physical significance of the basic block size $R_0$: it is a length scale, typically of the order of MRO length scales in glasses, beyond which $\langle Q^{-1}\rangle$ attains universal value (As discussed in Ref.^33^, $R_0$ is also the distance between two molecules at which two competing forces become equal in strength). Further $R_0$ is also consistent with another assumption made in our study i.e regarding the isotropy and homogeneity of the stress filed of the basic block; this follows because almost all molecules within a spherical block of radius $R_0$ are subjected to same interaction strength (with 8 molecules within a basic block). The omnipresence of dispersion forces indicates the application of our results to other disordered materials too.

The analysis presented here takes only dispersion type inter-molecular forces into account and neglects the induction forces which restricts, in principle, the application of our results to non-polar molecules. We believe however that inclusion of induction forces would only change numerical value of *b* (given by Eq. (26)) and would not affect the derivations given in “Super block: phonon mediated coupling of basic blocks” to “Discussion” sections. Similarly a generalization of the present theory by including electronic interactions may explain the universality in context of metallic glasses.

## Supplementary Information

Supplementary Information.
