# Supplementary material for: Universality of ultrasonic attenuation coefficient of amorphous systems at low temperatures
Source: Sci Rep. 2022 Feb 17;12:2662. doi: 10.1038/s41598-022-06589-7 (PMC8854728; doi:10.1038/s41598-022-06589-7)
Supplement: Supplementary file 1 — Supplementary Information. [file 41598_2022_6589_MOESM1_ESM.pdf]

Supporting information

I. STRUCTURAL UNITS OF THE GLASSES USED FOR M IN TABLE 2

The basic structural unit in a glass depends on the presence of various cations some of which act as network formers and others act as network modifiers (e.g. see pages 9-11 of [1]). Here we give the glass composition and the dominant structural units for 18 glasses used in tables I. The molar mass M_1 in Table I refers to the masses of these units.

(1) $a - SiO_2$: The 3-d network in this case has the basic structural unit is Si[SiO4] with mass $M_1 = 120.09$ (see page 37 of [2], section 11.4.1 of [8]), also see section 2.2 and fig2.7(a) of [3]).

(2) BK7: (wt %): The constituents in this case are 69.9 SiO_2, 9.9B_2O_3, 8.4NaO_2, 8.4K_2O, 2.5BaO. With 70% SiO_2, the basic structural unit in this case is [SiO4] [4] (also see section 11.4.1, 12.1 of [8]) with mass $M_1 = 60.08$.

(3) As_2S_3: The glass in this case forms chain like structure e.g. $S - S$, $As - As$ or $As - S$ (i.e S or As of one chain interacting with neighboring one). As S is dielectric, we use it as the basic unit participating in VWD interaction (see page 56 of [11], page 125 of [6], also see section 2.2 and fig 2.7(b) of [3]) and therefore choose $M_1 = 32$. Using molar weight of As for the purpose, gives $\mathcal{B}_l = 0.29, \mathcal{B}_t = 0.62$ and $\frac{\gamma_l}{c_l} = \frac{\gamma_t}{c_t} = 0.17$.

(4) LaSF7: Also known as dense lanthanum flint glass, it contains mostly B_2O_3, La_2O_3 and ThO_2 with a few % of Ta_2O_3 and Nb_2O_3: Here the first three are main net-forming components and last two are net modifiers As net formers are in equal proportion (with each of 30% weight-fraction), each one can play the role of structural unit. In this case, the structural units of each component is triangular i.e BO_3, LaO_3, ThO_2. The mass M_1 in this case is then obtained as follows: $M = \frac{30}{100}(BO_3 + LaO_3 + ThO_2)$.

(5) SF4: The glass composition in this case is 60.8SiO_2, 34.9PbO, 2.5K_2O and 1.8 other. with 61% SiO_2 but with 35% PbO, this has a basic structural unit is Si_2O_5 with its mass $M_1 =$ (due to compound of type $2SiO_2.PbO$, 2 Si atoms get coordinated with $5O$, (see page 17 of [9], page 14-15 of [5]).

(6) SF59: Here the constituents are 35.3.8SiO_2, 55.6PbO, 0.8K_2O, 7.9B_2O_3 and 0.4 other material. with reduced fraction of SiO_2, the compund is of form $2PbO.SiO_2$ leading to a basic structural unit of type SiO_4 tetrahedral with $M_1 =$ (see page 17 of [9], page 14-15 of

[5]).

(7) V52: The constituents are $57.8ZrF_4$, $33.8BaF_2$ and $8.5ThF_4$. Due to higher content of ZrF_4, the main structural unit in this case is ZrF_4 tetrahedral with main role of cations Ba and Th is to cause 2-d structure (network modifiers) (page 35 of [7], page 157 of [5]). Thus M_1 in this case is used as the molar mass of ZrF_4.

(8) BALNA: 52 ZrF_4, 24 BaF_2, 4 AlF_3 and 20 NaF. Due to higher content of ZrF_4, the main structural unit in this case is ZrF_4 tetrahedral with main role of the cations Ba, Al and Na is that of network modifier (i.e to cause 2-d structure) (page 35 of [4], page 157 of [7]). Thus M in this case is used as ZrF_4.

(9) LAT: 60 ZrF_4, 33 ThF_4, 7 LaF_3. Due to higher content of ZrF_4, the main structural unit in this case is ZrF_4 tetrahedral with main role of the cations Ba, Al and Na is that of network modifier (i.e to cause 2-d structure) (page 35 of [7], page 157 of [5]). Thus M in this case is used as ZrF_6.

(10) $a - Se$: Se atoms form chains or 8 atom rings through covalent/ionic bonding. The atoms on neighboring chains or rings interact by lone-pair electrons (VWD). So M is that of Se atom (page 43 of [11], page 115 of [6]).

(11) $Se_{75}Ge_{25}$: As both Ge and Se are network-formers, we use both atoms to calculate M_1 (page 115 of [6]). Thus $M_1 = M_2$

(12) $Se_{60}Ge_{40}$: Here again both Ge and Se act as network-formers (page 115 of [6]), M_1 is therefore obtained form both of them and therefore $M_1 = M_2$.

(13) $LiCl : 7H_2O$: The $LiCl$ molecule in presence of H_2O forms $Li(H_2O)Cl_3$ tetrahedral which seems to act as a basic structural unit. Thus M_1 used in our analysis corresponds to this unit .

(14) Zn-Glass: This glass consists of $60ZnF2$, $20BaF_2$, $20NaPo_3$. Due to higher content of ZnF_2, the main structural unit in this case is ZnF_2 with main role of the cations Ba and Na is that of network modifier (i.e to cause 2-d structure) [10]. Thus M_1 for this case is used as ZnF_2.

(15) PMMA: The monomer here has a phenyl group C_6H_5 which appears as a side unit while the units along the main chain strongly connected by covalent bonds. As VWD interaction occurs between molecules on different chains, the main unit playing role here is C_6H_5. So M_1 taken is that of C_6H_5.

(16) PS: The monomer here has a phenyl group C_6H_5 as a side unit as well as $CH = CH_2$

unit while the units along the main chain strongly connected by covalent bonds. As VWD interaction occurs between molecules on different chains, the main unit playing role here seems to be $CH - CH$ or $CH = CH_2$. The former could be part of Phenyl group. Note unlike other polymers, the monomer of PS is small and therefore only part of Phenyl group may be loosely held and participate in VWD.

(17) PC: as the monomer here is a big molecule, the Phenyl group may be loosely held and participate in VWD. So M_1 taken is that of C_6H_5

(18) ET1000: here again the monomer is a big molecule, the Phenyl group may be loosely held and participate in VWD. So here again M_1 taken is that of C_6H_5.

II. RELATION BETWEEN γ AND γ_m

Consider the linear response of a basic block, labeled as $''s''$ containing g_0 molecules, to an external strain field. The existence of long wavelength phonons at low temperatures leads to a phonon-mediated pair-wise interaction among molecules, decaying as inverse cube of distance between them. Consider two molecules, labeled as "1" and "2" with their centers at a distance r within the block. Following the same formulation as in case of blocks, and with $T_{\alpha\beta}$ as the stress tensor component for the molecule, the corresponding interaction energy can be written as

$$V_{stress}(\mathbf{r}) = \frac{1}{\alpha_0 \pi \rho_m c^2} \sum_{te} \frac{\kappa^{(12)}_{\alpha\beta\gamma\delta}}{\mid \mathbf{r}_1 - \mathbf{r}_2 \mid^3} \; T^{(1)}_{\alpha\beta} \otimes T^{(2)}_{\gamma\delta} \tag{1}$$

with $\alpha_0 = 4$, $r = |\mathbf{r}_1 - \mathbf{r}_2|$, $\kappa^{(12)}_{\alpha\beta\gamma\delta}$ as in the case of block-block interaction (given by eq.(**??**)), ρ_m as the mass-density of the molecule, c as speed of the sound waves. The ensemble averaged interaction energy can then be approximated as

$$V_{stress}(r) \approx \frac{\gamma_m^2}{\rho_m \; c^2 \; r^3} \tag{2}$$

with γ_m as the average strength of the phonon induced r^{-3} coupling of the two molecules.

The interaction parameter γ_m can be determined as follows. As $\mathrm{Tr}(V_{stress}) = 0$, one can write, with $\langle . \rangle_e$ as the ensemble average

$$\langle \mathrm{Tr}(V^2_{stress}) \rangle_e \approx \left(\frac{\gamma_m^2}{\rho_m \; c^2 \; r^3} \right)^2 \tag{3}$$

But

$$\langle \mathrm{Tr}(V_{stress}^2)\rangle_e \approx \left(\frac{1}{\alpha_0 \pi \rho_m c^2 \, r^3}\right)^2 \sum_{te,te'} \kappa^{(12)}_{\alpha\beta\gamma\delta} \, \kappa^{(12)}_{\alpha'\beta'\gamma'\delta'} \, \langle \mathrm{Tr}\left(T^{(1)}_{\alpha\beta} \, T^{(2)}_{\gamma\delta} T^{(1)}_{\alpha'\beta'} \, T^{(2)}_{\gamma'\delta'}\right)\rangle_e \tag{4}$$

Further as

$$\mathrm{Tr}\left(T^{(1)}_{\alpha\beta} \, T^{(2)}_{\gamma\delta} T^{(1)}_{\alpha'\beta'} \, T^{(2)}_{\gamma'\delta'}\right) = \sum_{n,m,k,l} T^{(1)}_{\alpha\beta;nm} \, T^{(2)}_{\gamma\delta;mk} T^{(1)}_{\alpha'\beta';kl} \, T^{(2)}_{\gamma'\delta';ln} \tag{5}$$

with $T^{(1)}_{\alpha\beta;nm} \equiv \langle n|T^{(1)}_{\alpha\beta}|m\rangle$, state $|n\rangle$ referring to one of the $\mathcal{N}$ single molecule states (unperturbed). Following similar ideas as in the case of a block, we have $\langle T^{(1)}_{\alpha\beta;mn} T^{(2)}_{\alpha\beta;kl}\rangle = 0$ $\forall m, n, k, l$ and $\langle T^{(1)}_{\alpha\beta;mn} T^{1)}_{\alpha'\beta';kl}\rangle = \tau^2 \; \delta_{\alpha\alpha'}\delta_{\beta\beta'}(\delta_{nk}\delta_{ml} + \delta_{nl}\delta_{mk})$. On ensemble average, the above leads to

$$\begin{aligned} \langle \mathrm{Tr}\left(T^{(1)}_{\alpha\beta} \, T^{(2)}_{\gamma\delta} T^{(1)}_{\alpha'\beta'} \, T^{(2)}_{\gamma'\delta'}\right)\rangle &= 2 \sum_{m,n} \langle \left(T^{(1)}_{\alpha\beta;nm}\right)^2\rangle \, \langle \left(T^{(2)}_{\gamma\delta;mn}\right)^2\rangle \; \delta_{\alpha\alpha'}\delta_{\beta\beta'}\delta_{\gamma\gamma'}\delta_{\delta\delta'} &(6)\\ &= 2 \, \mathcal{N}^2 \, \tau^2 \; \delta_{\alpha\alpha'}\delta_{\beta\beta'}\delta_{\gamma\gamma'}\delta_{\delta\delta'} &(7) \end{aligned}$$

The above on substitution in eq.(3) leads to

$$\mathrm{Tr}(V_{stress}^2) \approx 2 \left(\frac{\mathcal{N} \, \tau^2}{\alpha_0 \pi \rho_m c^2 \, r^3}\right)^2 \sum_{te} (\kappa^{(12)}_{\alpha\beta\gamma\delta})^2 \tag{8}$$

Comparison of eq.(8) with eq.(3) gives

$$\gamma_m^2 \approx \frac{\mathcal{N} \, \tau^2}{\alpha_0 \pi} \, K \, \sqrt{2} \tag{9}$$

where $K^2 = \sum_{te} (\kappa^{(12)}_{\alpha\beta\gamma\delta})^2$.

To relate the above to basic block property γ^2, we proceed as follows. The stress-operator for a basic block can be written in terms of those of molecules:

$$\Gamma^{(s)}_{\alpha\beta;mn} = \sum_{a=1}^{g_0} T^{(a)}_{\alpha\beta;mn}.$$

The subscripts m, n now refer to an arbitrary pair chosen from $N = \mathcal{N}^{g_0}$ many body states of the basic block (e.g. the product states $|e^0_m\rangle$ and $|e^0_n\rangle$ of single molecule states). Further $T^{(x)}_{\alpha\beta;mn} \neq 0$ only if $|e^0_m\rangle$ and $|e^0_n\rangle$ differ only by the contribution from the xth molecule; this leaves only $\mathcal{N}^{g_0+1}$ non-zero matrix elements for each $T^{(x)}$. Noting that stress marix elements of different molecules are uncorrelated, it can now be shown that

$$\sum_{m,n=1}^{N} \langle \Gamma^{(s)}_{\alpha\beta;mn} \, \Gamma^{(s)}_{\alpha\beta;nm}\rangle_e = \sum_{m,n=1}^{N} \langle \left(T^{(a)}_{\alpha\beta;mn}\right)^2\rangle_e = g_0 \, \mathcal{N}^{g_0+1} \, \tau^2 = g_0 \, N \, \mathcal{N} \, \tau^2. \tag{10}$$

Further assuming homogeneous interaction within a basic block, the variances of all matrix elements of the basic block can be approximated as almost equal. The left side of eq.(10) is then equal to $N^2\,\nu^2$ (with $\langle(\Gamma^{(s)}_{\alpha\beta;mn})^2\rangle_e = \nu^2$) which leads to

$$\gamma^2 = g_0\,\mathcal{N}\,\tau^2 = \frac{g_0\alpha_0\pi}{K\sqrt{2}}\,\gamma_m^2 \tag{11}$$

Taking $\kappa^{(12)}_{\alpha\beta\gamma\delta}$ from eq.(8) of main text, we have $K^2 = 18\left[1+4\left(1-\frac{c_t^2}{c_l^2}\right)\right]$

---

[1] D.Hulsenberg, A. Harnisch, A. Bismarck, **Microstructuring of Glasses**, ed. Springer, (2008).

[2] W.Vogel, **Glass Chemistry**, 2nd Ed.,Springer-Verlag, (1994).

[3] K. Tanaka and K. Shimakawa, **Amorphous Chalcogenide semiconductors and Related Materials**, ed. Springer, (2011)

[4] L.D. Pye, V.D.Frechette and N.J.Kreidl, **Borate Glasses** ed. Plenum Press (N.Y.) (1978).

[5] M.H.Lewis, **Glasses and Glass-ceramics**, ed. Chapman and Hall (London, N.Y.), (1989).

[6] Z. Borisava, **Glassy Semiconductors**, Springer, (1981).

[7] P.W.France et.al., **Fluoride Glass Optical Fibres**, ed. CRC Press (U.S.A), (1990).

[8] J. Sestak, J.J.Mares and P.Hubik, **Glassy, Amorphous and nano-crystalline materials**, ed. Springer, (2011).

[9] H. Scholze, **Glass: nature, structure and properties**, Springer-Verlag, (1991).

[10] S. Aasland and T. Grande, S t r u c t u r e of Fluorozirconate Glasses and Melts*Chem. Papers 52, 21-28, (1998).

[11] A. Popov, **Disordered Semiconductors**, Taylor and Francis, 2nd ed. (2018).

[12] M.J. Weber, **Handbook of optical materials**, CRC Press (U.S.A), (2003).
